# Management of Pulmonary Artery Aneurysms in Pulmonary Hypertension: A Single-Center Review of 3 Cases

**DOI:** 10.1155/2019/1924014

**Published:** 2019-10-09

**Authors:** Talha Ahmed, Gautam V. Ramani, Mehrdad Ghoreishi, Ayesha Safdar, Bartley P. Griffith

**Affiliations:** ^1^Department of Medicine, University of Maryland Midtown Campus, USA; ^2^Department of Medicine, University of Maryland, USA; ^3^Department of Surgery, University of Maryland, USA; ^4^Army Medical College, Pakistan

## Abstract

Pulmonary artery aneurysms (PAAs) are defined as having pulmonary artery diameter of greater than 40 mm. PAAs are rare and can occur in various pulmonary diseases. There are no clear-cut guidelines regarding the management of PAAs, and recommendations for management are made based on expert consensus opinion, case reports, and institutional experience. This series highlights three patients with pulmonary hypertension (PH) and PAA. The clinical course and diagnostic findings and the decision-making involved in the treatment are reviewed. An overview of three distinct management strategies including medical management, heart/lung transplant, and surgical aneurysm repair is presented.

## 1. Case 1

**Figure 1:**
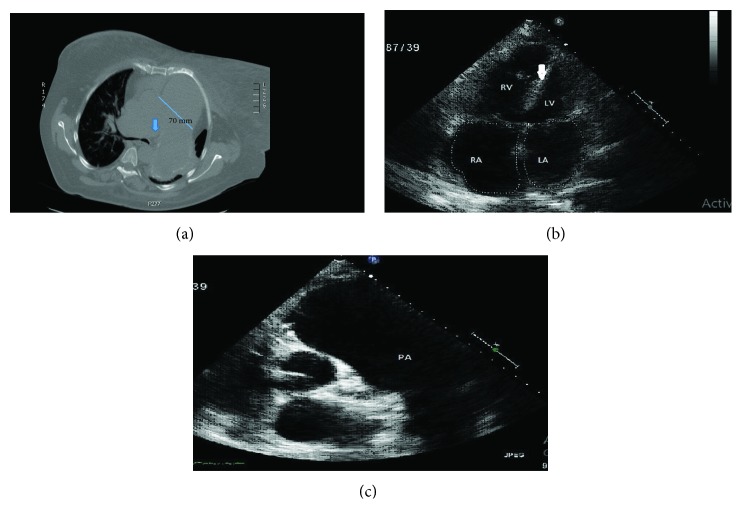
(a) Noncontrast chest CT showing main pulmonary artery aneurysm measuring 70 mm with blue arrow pointing the possible intimal flap. Rapid tapering of pulmonary vessels can be appreciated on the right lung zones. (b) Transthoracic echocardiogram apical four-chamber view showing severely dilated right ventricle and septal flattening (white arrow). (c) Transthoracic echocardiogram parasternal short axis view at the level of aortic valve showing severe dilation of pulmonary artery.

A 51-year-old female with long-standing pulmonary hypertension (PH) on sildenafil, suspected to be pulmonary arterial hypertension (PAH) with known prior history of pulmonary artery aneurysm (PAA) but was lost to follow-up, presented to the hospital with hemoptysis. She suffered from profound anxiety and often missed appointments due to fear of leaving the home. Computerized tomography (CT) scan of the chest showed an aneurysm of the main pulmonary artery (PA) measuring 85 mm in diameter with an intimal flap concerning for pulmonary artery dissection (Figure 1(a)). Transthoracic echocardiogram (TTE) revealed a hypertrophied, moderately dilated, and dysfunctional right ventricle (RV) with an estimated right ventricular systolic pressure (RVSP) of 78 mmHg and severe dilation of main PA compatible with severe PH (Figures 1(b) and 1(c)). Due to the massive size of the PAA with suspected dissection, PA catheterization was deferred. She was evaluated for lung and lung/heart transplant but was not deemed a suitable candidate due to her significant psychiatric history. Her risks of surgical or percutaneous repair were felt to be prohibitive due to the degree of PH and RV dysfunction. A decision was made to manage her medically, and she was started on intravenous treprostinil which was rapidly uptitrated. This was done as a palliative measure for symptomatic improvement of her PH which was suspected to be most likely idiopathic as well as the fact that the case posed limited diagnostic and therapeutic evaluation. Her functional status improved after a few months of parenteral treprostinil from WHO Functional Class (FC) III to FC II. Routine follow-up studies including 6-minute walk test (6MWT), pulmonary function tests (PFTs), and repeat TTE were not performed primarily due to patient anxiety. She was found deceased on the ground three years after the hospital admission most likely as a complication of PAA rupture, though no autopsy was performed.

## 2. Case 2

**Figure 2:**
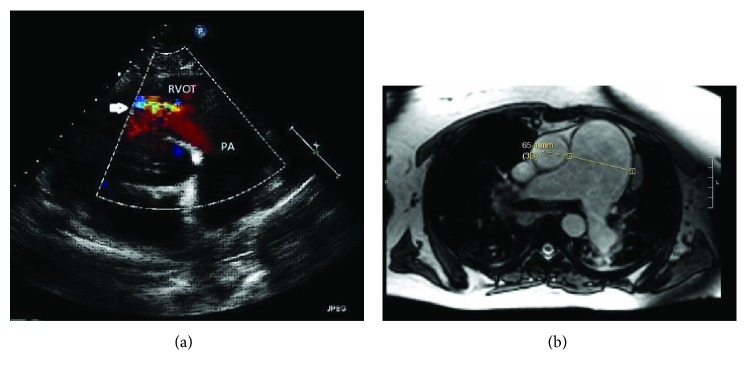
(a) Transthoracic echocardiogram parasternal short axis color Doppler showing severe pulmonic regurgitation (white arrow) and dilated pulmonary artery. (b) Cardiac MRI showing marked aneurysmal enlargement of the main PA.

A 57-year-old female with a long-standing history of fibrocystic sarcoidosis developed PH 18 years following her initial diagnosis. A CT scan of the chest revealed findings compatible with advanced pulmonary sarcoidosis with primarily apical involvement, bilateral hilar lymphadenopathy, and PH with aneurysmal dilation of main PA to 65 mm. Right heart catheterization (RHC) showed elevated right-sided filling pressures with a mean PA pressure (mPAP) of 46 mm Hg, normal pulmonary capillary wedge pressure (PCWP) of 12 mmHg, and pulmonary vascular resistance (PVR) of 7.1 Wood units. TTE revealed severe dilation with dysfunction of the RV and severe pulmonary valvular regurgitation (PVR) (Figure 2(a)). Despite the etiology of WHO Group 5 PH, she was started on sildenafil and macitentan given the severity of her symptoms. Inhaled treprostinil was added later due to persistent dyspnea. Her symptoms and supplemental oxygen requirement did not improve, and she was admitted for clinical worsening. A cardiac MRI was performed. The study did not reveal findings of cardiac sarcoidosis; however, it did reveal PAA and severe pulmonic regurgitation (Figure 2(b)). PFTs showed severe restrictive lung disease (FEV1 of 55%, FVC of 44% with FEV1/FVC of 126%). Preoperative ventilation/perfusion (V/Q) scan showed heterogeneous ventilation as well as perfusion activity in both lungs but with no V/Q mismatch. She was considered for bilateral lung transplant due to severe and progressive pulmonary sarcoidosis refractory to medical management (steroids) as well as severe and progressive PH. However, due to coexisting PAA and severe PVR, it was decided to proceed with heart-lung transplantation. Surgery was performed under venoarterial extracorporeal membrane oxygenation (VA-ECMO) support. A 7 cm proximal PA aneurysm was found. After achieving hemostasis by clamping the aorta, the heart was removed followed by the removal of PA intrapericardially till the bifurcation, and then, the anterior segments of the right and left PA were resected leaving the posterior segment behind followed by intrapericardial stapling of pulmonary veins. This was followed by lung removal and successful placement of the heart and lung into the pericardial and pleural space, respectively, with anastomosis to the pulmonary vessels. She was successfully decannulated from VA-ECMO and had an unremarkable postoperative course. At 2-year follow-up, the patient had marked improvement of symptoms, was walking 3 miles a day on treadmill, and had a normal surveillance TTE and PFTs (FEV1 of 86%, FVC of 89% with FEV1/FVC of 102%).

## 3. Case 3

A 73-year-old female was referred from an outside hospital with worsening edema, dyspnea, and concern for acute pulmonary embolism (PE). On presentation to the outside hospital, she was hypoxic with asymmetric lower extremity swelling. The lower extremity venous Doppler did not reveal any evidence of deep venous thrombosis (DVT).

**Figure 3:**
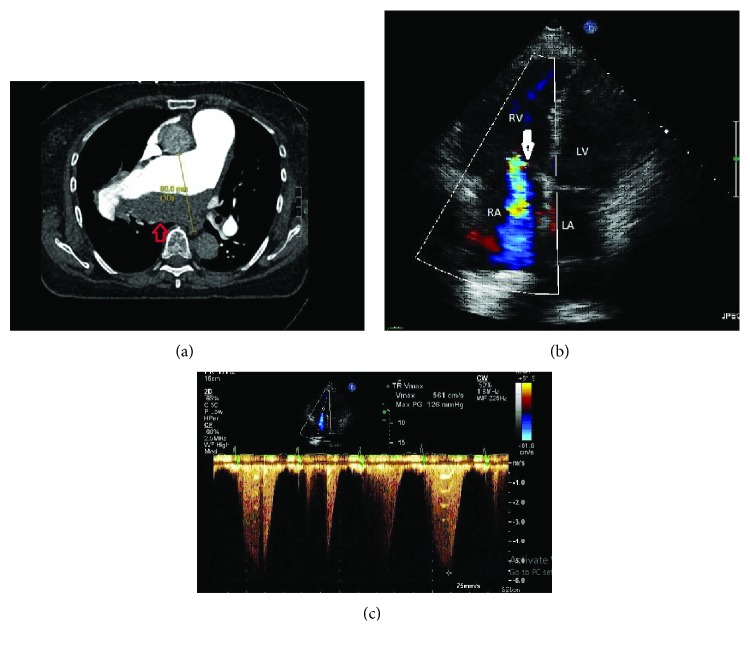
(a) CTA chest showing right pulmonary artery aneurysm measuring 80 mm in the greatest dimension with laminated thrombus in the aneurysmal sac (red arrow). (b) Transthoracic echocardiogram apical four-chamber view showing dilated RV, severe TR (white arrow), and systolic septal flattening consistent with RV pressure overload. (c) Extremely elevated estimated PA systolic pressure measurements to 134 mmHg indicating severe PH.

Computerized tomography angiography (CTA) of the chest revealed a right PAA measuring 80 mm in diameter with laminated clot burden and compression of the right bronchus (Figure 3(a)). There was a question of acute PE vs. chronic thromboembolic pulmonary disease (CTED) vs. in situ thrombus within the aneurysm sac. She was started on intravenous heparin. A careful review of the images, however, did not reveal classic findings of CTED, and there was a suspicion for PAH and associated PAA with clot formation in the aneurysmal sac. A V/Q scan showed low probability for pulmonary embolism. TTE showed right ventricular dysfunction with elevated RVSP of 134 mmHg and severe tricuspid regurgitation (TR) (Figures 3(b) and 3(c)). RHC was deferred due to the presence of the aneurysm. PFTs revealed a restrictive pattern with FEV1 of 32%, FVC of 28%, and FEV1/FVC of 115%. Owing to significant bronchial compression by the massive aneurysm and profound PA dilatation, a decision was made to proceed with surgical repair. During surgery, a large deeply hidden aneurysm situated just at the bifurcation of the main pulmonary trunk was opened and found full of clotted blood. There was evidence of dissection of aneurysm involving both right and left PAs. She underwent aneurysmectomy and homograft repair, and her postoperative course was unremarkable. On subsequent outpatient visits, patient's symptoms improved from WHO FC III at the time of presentation to WHO FC II, but RV dilatation/dysfunction persisted on TTE. An outpatient RHC was completed which revealed mPAP of 45 mmHg and increased PVR to 10 Wood units compatible with PAH. She was subsequently started on tadalafil and ambrisentan with plan for lifelong anticoagulation. At 9-month follow-up, the patient had improvement in her symptoms and could walk up and down a 1-2 flight of stairs (WHO FC II) and walked 425 m (98% of predicted) during 6MWT on room air with improvement of RV size and function on TTE.

## 4. Discussion

PAAs are not routinely encountered in clinical practice and can occur in a spectrum of pulmonary and systemic diseases. The true incidence of PAAs remains unclear in the general population, but in 1947, Deterling and Clagett reported eight cases in a series of more than 100,000 autopsies (0.00008%) [1]. Risk factors for PAA include increasing age, male sex, elevated mPAP, duration of PH, vasculitis, and connective tissue diseases [2]. In patients with PH, regardless of etiology, the incidence is significantly higher (1.25%) [2].

The normal diameter of the PA on CT ranges 25 mm ± 3 mm with 29 being the upper limit of normal. For the left and right PAs, the upper range of normal is 20 mm. Most of the studies have defined a PAA as a main PA > 40 mm.

The course of patients with isolated PAA is clinically less severe than the patients with an underlying cardiopulmonary disease including PH, congenital heart disease, and infections such at tuberculosis and syphilis (which are rare in the western world). Some of the high-risk features of PAA include PA diameter exceeding 75 mm, rapid pulmonary artery diameter growth (>2 mm/year), and high mean PA pressures on RHC (exceeding 50 mmHg) [3]. Common complications of PAAs include in situ thrombosis, pulmonary embolism, compression of adjacent structures including left main coronary artery as well as adjacent bronchi, and dissection of the pulmonary artery [4]. PAAs can progress to rupture, and one-third of all the reported cases of PAA died of complications resulting from rupture including lethal hemoptysis, asphyxiation, exsanguination, and sudden death. There are no established guidelines for the management of PAAs, and treatment usually is individualized. Surgical repair is usually recommended for large (>60 mm) PAAs due to the risk of rupture or dissection. These criteria are however based on case studies [5, 6]. Other potential indications include development of symptoms related to bronchial compression, thrombus formation in the aneurysm sac, and evidence of valvular pathology such as severe pulmonic regurgitation. In case of pulmonary valve insufficiency, timing of surgical intervention is determined by changes in RV size and function [7]. Results from previous case reports have also demonstrated improvements in lung function and PH after surgical repair [8]. Data from case reports and series in the past also suggest that treating PH with vasodilators does not affect the growth rate of PAAs. Lung transplant is an option when significant parenchymal or progressive pulmonary vascular disease is present [9].

Various surgical management options have been described in the literature. Aneurysmectomy with homograft repair is being increasingly utilized, especially when the aneurysm is compressing adjacent structures, and PA pressures are manageable [10]. The third patient in our series did well after surgical repair indicating that despite the presence of PAH, with an experienced team, good surgical outcomes are feasible. Angioplasty with plication, reconstruction of the main pulmonary artery with autologous or prosthetic tissue has been reported in the literature in the absence of underlying PH [11]. Endovascular coiling and embolization of peripheral PAA have also been reported in some cases in the past [12].

In this case series, we highlight three distinct approaches to PAA management in PH. These include medical management alone with pulmonary arterial vasodilators, transplant including lung or heart/lung transplant, and surgical resection (aneurysmectomy). Each of these options has its strength and weakness, and case-based management is usually considered based on individual patient profile.
